# Mastoid cortex osteoma associated with middle-ear and mastoid cholesteatoma: a case report

**DOI:** 10.25122/jml-2026-0069

**Published:** 2026-06

**Authors:** Maria Denisa Zica, Catalina Voiosu, Andreea Rusescu, Razvan Hainaorsie, Viorel Zainea

**Affiliations:** 1 Department of Otorhinolaryngology, Carol Davila University of Medicine and Pharmacy, Bucharest, Romania; 2 Institute of Phonoaudiology and Functional ENT Surgery, Professor Dr. D. Hociota, Bucharest, Romania

**Keywords:** osteoma, mastoid cortex, cholesteatoma, temporal bone, Eustachian tube dysfunction, DWI-MRI, canal wall-down mastoidectomy, case report, CWD, canal wall-down, CWU, canal wall-up, DWI-MRI, diffusion-weighted magnetic resonance imaging, EAC, external auditory canal, HRCT, high-resolution computed tomography, IAC, internal auditory canal, ME, middle ear, non-EPI, non-echo-planar imaging, TM, tympanic membrane

## Abstract

Osteomas of the temporal bone are rare benign tumors that, in a small minority of cases, may give rise to secondary cholesteatoma. We report a case in which two independent pathological mechanisms appeared to coexist, resulting in a configuration not previously described in the literature. A 51-year-old woman presented with one year of right-sided otorrhoea and progressive mixed hearing loss unresponsive to antibiotic therapy. High-resolution computed tomography (HRCT) demonstrated a giant sessile osteoma arising from the anterior mastoid cortex with complete external auditory canal (EAC) obstruction. Non-echo-planar diffusion-weighted MRI (non-EPI DWI-MRI) identified cholesteatoma restricted to the middle ear and mastoid antrum, clearly distinct from chronically inflamed mastoid air cells. Intraoperatively, a near-intact tympanic membrane with a small attic retraction pocket formed the anterior boundary; no canal-wall cholesteatoma was present. Canal wall-down (CWD) radical mastoidectomy with osteoma excision and meatoconchoplasty achieved complete disease eradication. Histopathology confirmed compact osteoma and cholesteatoma. The cavity was dry and stable at the three-month follow-up. Upon review of the published case reports of temporal bone osteoma with associated cholesteatoma, no previously reported case combined a giant osteoma of the anterior mastoid cortex with cholesteatoma confined entirely to the middle ear and mastoid behind a structurally preserved tympanic membrane. The intraoperative finding of an attic retraction pocket suggests that Eustachian tube dysfunction may have played an independent contributory role alongside the osteoma. Combined HRCT and non-EPI DWI-MRI was essential for preoperative characterization and surgical planning.

## Introduction

Osteoma of the temporal bone is an uncommon benign tumor of mature lamellar bone, with an estimated prevalence of 0.05% among patients presenting for otological surgery [1,2]. Approximately 20% of temporal bone osteomas arise from the mastoid cortex rather than from the tympano-mastoid or tympano-squamous suture lines [3-5]. Their clinical course is usually indolent; however, large lesions may obstruct the external auditory canal (EAC), impair centrifugal keratinocyte migration, and, in a small but clinically important minority, precipitate secondary cholesteatoma [6,7]. The published case reports of this association — beginning with Orita *et al*. in 1998 [8] and including the subsequent reports by Lee *et al*. [9], Viswanatha [10], Domínguez Pérez *et al*. [11], Abhilasha and Viswanatha [5], laccarino *et al*. [12], and Dosemane *et al*. [2] — consistently describe cholesteatoma in this setting, involving the EAC canal wall as its primary or extending site of disease. This case report was prepared in accordance with the CARE (CAse REport) guidelines [13].

The case reported here presented two features that, upon review of the available published literature, do not appear to have been previously described together: osteoma arising from the mastoid cortex, and secondary cholesteatoma confined entirely to the middle ear and mastoid without any EAC canal-wall component, bounded anteriorly by a near-intact tympanic membrane. This configuration was not identified in the reviewed literature and may reflect an alternative pathogenic mechanism. We present the clinical, radiological, surgical, and histopathological findings and discuss the implications for diagnosis and management.

## Case presentation

### Clinical history

A 51-year-old woman presented with a one-year history of persistent right-sided otorrhoea and progressive mixed hearing loss that had not responded to a full course of topical fluoroquinolone and systemic antibiotic therapy. Microbiological culture of the aural discharge identified *Pseudomonas aeruginosa*. She denied any history of head trauma, cold-water exposure, prior ear surgery, or relevant comorbidities. Otoscopy revealed complete right EAC stenosis with mucopurulent secretions; the left ear was entirely normal. Tuning fork tests were consistent with right-sided mixed hearing loss.

### Audiological assessment

**Figure 1:**
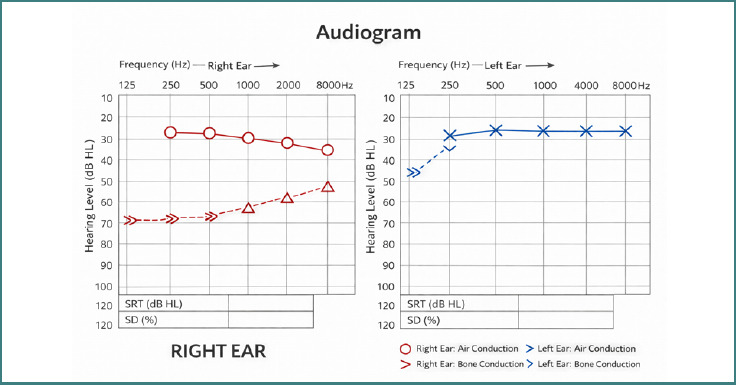
Pure-tone audiogram: moderate mixed hearing loss in the right ear (red) with a persistent air-bone gap across all frequencies, indicating both conductive and sensorineural components. The left ear (blue) demonstrates normacusia.

Pure-tone audiometry demonstrated moderate mixed hearing loss in the right ear with a persistent air-bone gap across all tested frequencies, and normacusia in the left ear (Figure 1). Mean pure-tone average (0.5–4 kHz) was 48 dB HL air-conduction and 28 dB HL bone-conduction, yielding an air-bone gap of 20 dB. The sensorineural component was disproportionate to simple canal obstruction, raising the possibility of chronic middle-ear pressure effects on cochlear function and supporting the rationale for obtaining magnetic resonance imaging (MRI) in addition to high-resolution computed tomography (HRCT).

### Radiological workup

**Figure 2:**
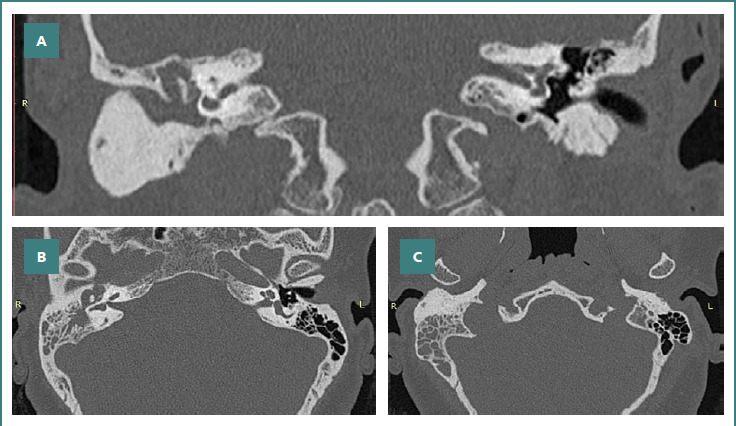
High-resolution computed tomography findings of the right temporal bone. A, Axial section showing a sessile osteocondensing mass arising from the anterior mastoid cortex, with complete opacification of the external auditory canal lumen and tympanic cavity. B, Coronal lateral section showing extension of the osteoma toward the mastoid apex, with scutum erosion and dense antral opacification. C, Coronal medial section showing preserved medial external auditory canal caliber, confirming that the stenosis originates from the lateral cortical osteoma.

HRCT of the temporal bones without contrast demonstrated a large, sessile, osteocondensing lesion arising from the anterior mastoid cortex and extending caudally to the apex. At its cranial pole, it narrowed the lateral half of the EAC; the medial EAC retained normal caliber. The tympanic cavity, mastoid antrum, and mastoid air cells were uniformly opacified. Significant erosion of the scutum, incus head, body, and short process was evident, strongly suggesting cholesteatoma. No tegmen dehiscence or sigmoid sinus exposure was identified (Figure 2A–C).

**Figure 3:**
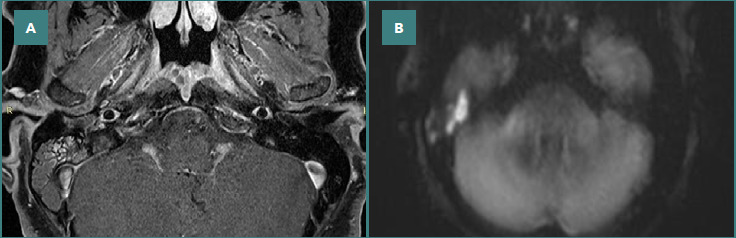
Magnetic resonance imaging findings of cholesteatoma and associated inflammatory changes. A, Axial non-EPI DWI showing restricted diffusion (high signal) in the deep EAC, tympanic cavity, and mastoid antrum, confirming cholesteatoma; the mastoid air cells lacked restricted diffusion, establishing clear compartmentalization. B, Axial T1-weighted postgadolinium sequence showing absent enhancement in the tympanic cavity, distinguishing cholesteatoma from granulation tissue; moderate enhancement of the external auditory canal wall reflects chronic otitis externa.

MRI with gadolinium contrast, using a 1.5-T non–echo–planar diffusion-weighted imaging (non-EPI DWI) protocol, provided definitive soft-tissue characterization. Material in the deep EAC, tympanic cavity and recesses, and mastoid antrum demonstrated restricted diffusion with absent gadolinium enhancement, characteristic of cholesteatoma, for which non-EPI DWI achieves sensitivity and specificity exceeding 90% [14]. The mastoid air cells showed a heterogeneous signal with peripheral rim enhancement, consistent with chronic inflammatory granulation tissue, and were clearly distinct from the cholesteatoma compartments. By establishing that cholesteatoma was confined to the middle ear and antrum while the mastoid air cells contained only inflammatory tissue, DWI-MRI directly shaped the surgical strategy and supported the decision to proceed with CWD rather than a more conservative approach (Figure 3AB).

### Surgical technique

**Figure 4:**
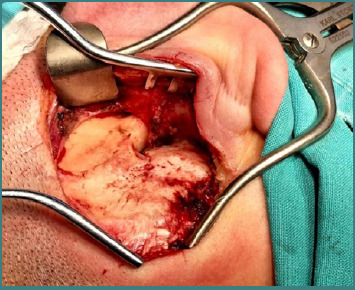
Retroauricular exposure: the giant mastoid cortex osteoma (asterisk) after periosteal elevation, extending from the anterosuperior mastoid to the mastoid apex

**Figure 5:**
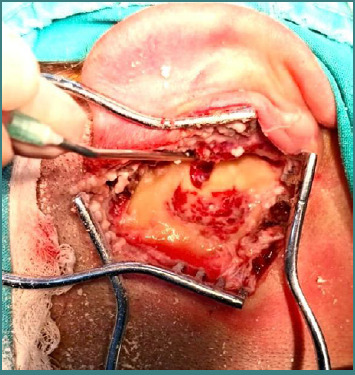
Post-canalplasty: the near-intact tympanic membrane (arrow) with a small attic gap following clearance of inflammatory material from the EAC. This finding indicates that cholesteatoma formed without direct canal epithelial migration.

**Figure 6:**
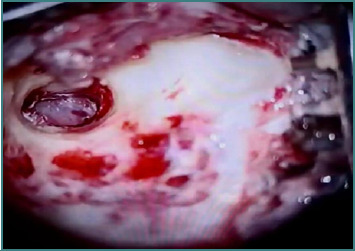
Osteoma excision in progress: diamond-burr drilling under continuous irrigation restores EAC caliber

Surgery was performed under general anesthesia via a retroauricular approach with continuous intraoperative facial nerve electromyographic monitoring throughout. Following periosteal elevation, the giant osteoma was apparent, occupying the anterosuperior mastoid cortex and extending to the apex (Figure 4). The linea temporalis and Macewen triangle served as anatomical landmarks. Chronic inflammatory material was cleared from the EAC, revealing a near-intact tympanic membrane; complete osteoma excision by diamond-burr drilling and formal canalplasty was performed (Figures 5 and 6).

**Figure 7:**
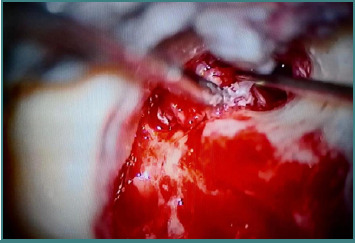
CWD radical mastoidectomy in progress: cholesteatoma matrix (arrows) in the mastoid antrum; facial buttress partially reduced; tympanic membrane (TM) intact anteriorly.

**Figure 8:**
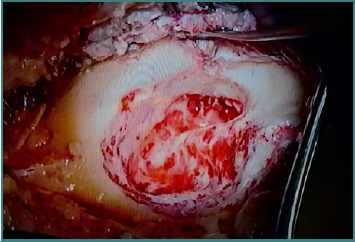
Completed radical mastoidectomy cavity: skeletonized tegmen (T), sigmoid sinus plate (S), facial ridge (F), and posterior semicircular canal (PSC); meatoconchoplasty opening (M).

Opening the mastoid antrum disclosed a large cholesteatoma consistent with the DWI-MRI localization. A CWD radical mastoidectomy was performed: the posterior EAC wall was progressively drilled to create a single externalized cavity; the facial ridge was thinned under continuous nerve monitoring; both incus head and malleus head were excised. After complete osteoma excision, a small attic retraction pocket containing the cholesteatoma matrix was directly visualized - an intraoperative finding consistent with retraction rather than direct canal epithelial migration as the route of entry. The cholesteatoma was strictly confined to the middle ear and mastoid; the tympanic membrane formed the anterior boundary throughout. A wide meatoconchoplasty completed the procedure. No intraoperative complications occurred (Figures 7 and 8).

### Histopathology

Microscopy of the bony specimen confirmed compact osteoma: mature lamellar bone with sparse fibrovascular channels, covered by fibrous periosteum, without mitosis or atypia [15]. Middle ear and antrum tissue demonstrated lamellated keratinous debris within stratified squamous epithelium, diagnostic of cholesteatoma [16,17]. Mastoid air cell tissue showed only chronic inflammatory granulation tissue without cholesteatoma matrix, fully corroborating the DWI-MRI compartmentalization. No malignant features were identified.

### Postoperative course

The early postoperative course was uneventful. At three weeks, the wound was well healed, and the mastoidectomy cavity showed healthy epithelialization without secondary infection. By three months, the cavity was completely dry and stable; otoscopy confirmed no residual or recurrent cholesteatoma. The patient reported no vestibular symptoms and described a marked reduction of the chronic otorrhoea and aural fullness that had been present preoperatively. Formal post-operative pure-tone audiometry was planned for the 12-month surveillance visit. Whether the sensorineural threshold will fully recover, partially recover, or remain stable will be determined at that point, and the result may provide additional evidence for the proposed mechanism.

Long-term surveillance includes repeat pure-tone audiometry, native HRCT to assess bony cavity stability, and non-EPI DWI-MRI at 12–18 months to screen for residual or recurrent cholesteatoma.

## Discussion

A literature search of PubMed using the terms “temporal bone osteoma” AND “cholesteatoma” identified all published case reports describing this association, beginning with the earliest documented case published by Orita *et al*. in 1998 [8] and including subsequent reports by Lee *et al*. [9], Viswanatha [10], Domínguez Pérez *et al*. [11], Abhilasha and Viswanatha [5], Iaccarino *et al*. [12], Mahalle [6], Hu *et al*. [7], and Dosemane *et al*. [2]. This search did not identify any case in which cholesteatoma was confined entirely to the middle ear and mastoid, without an EAC canal-wall component and with a structurally preserved tympanic membrane. In the reviewed cases, cholesteatoma was confined to the EAC canal wall in the majority, reflecting obstructed centrifugal epithelial migration as the dominant mechanism; a minority showed extension to the middle ear via tympanic membrane erosion or perforation; and one case reached the intracranial compartment, requiring subtotal petrosectomy [12]. The most comparable previously reported case is that of Domínguez Pérez *et al*. [11], who described a pedunculated osteoma of the aditus ad antrum associated with chronic cholesteatomatous otitis media; however, in that report, the osteoma was small and antral rather than a giant lesion of the anterior mastoid cortex, and the tympanic membrane and EAC patency were not characterised in sufficient detail to allow direct comparison. The configuration observed in the present case — a giant anterior-cortex osteoma with cholesteatoma confined entirely behind a structurally preserved tympanic membrane, with an attic retraction pocket as the proposed entry point — was not identified in the reviewed literature.

We propose that two independent pathological mechanisms coexisted in this patient, and that their simultaneous presence may explain the observed configuration. The mastoid cortex osteoma caused complete EAC obstruction, thereby preventing centrifugal keratinocyte migration, the mechanism underlying canal-wall cholesteatoma in all previously reviewed cases [6,7,16,17]. Paradoxically, the osteoma may have eliminated the canal-wall pathway rather than creating it. Concurrently, and independently of the osteoma, Eustachian tube dysfunction [18,19], whose primary etiology remains uncertain in this patient, as no nasopharyngeal pathology was identified, may have produced chronic negative middle-ear pressure, progressive pars flaccida retraction, and epithelial ingress via the attic retraction pocket observed intraoperatively — a mechanism consistent with the classical pathway of primary acquired cholesteatoma [20]. Neither mechanism alone would readily explain this presentation: without the osteoma, a conventional primary acquired middle-ear cholesteatoma might have developed; without the Eustachian tube dysfunction, the osteoma might have precipitated the canal-wall pattern documented in prior reports. Whether these mechanisms were truly independent or interrelated cannot be established from a single observation, and this question warrants attention in future reports. The interpretation is supported by three observations: the direct visualization of an attic retraction pocket containing the cholesteatoma matrix, the complete absence of canal-wall cholesteatoma, and a mixed audiometric pattern, in which the sensorineural component suggests chronic cochlear pressure effects inconsistent with isolated canal obstruction [21]. The compact osteoma histological subtype [15], growing more slowly than cancellous variants, may also explain how the lesion reached a giant size before becoming symptomatic, allowing a prolonged period of potential middle-ear pressure change.

Accurate preoperative imaging was essential and illustrates why both modalities are required in symptomatic temporal bone osteoma. HRCT confirmed the extracanalicular cortical origin, a finding that mandated the retroauricular approach from the outset, and identified scutum erosion and ossicular destruction [1,3,4]. Non-EPI DWI-MRI provided complementary soft-tissue characterization, distinguishing cholesteatoma from inflammatory mastoid content with sensitivity and specificity exceeding 90% [14], and directly shaped the surgical strategy by supporting the choice of a canal wall-down (CWD) over a canal wall-up (CWU) mastoidectomy. Its capacity to reduce the need for second-look surgery after CWU mastoidectomy [22] further supports its routine incorporation into preoperative and surveillance protocols for all cases in which soft-tissue opacification accompanies EAC osteoma [23].

The surgical approach should be individualized according to osteoma size, site of origin, and the extent of secondary disease. For small pedunculated EAC osteomas without cholesteatoma, transmeatal excision is effective, and a meta-analysis by Argaman *et al*. found no additional benefit from stalk drilling [24]. When canal-wall cholesteatoma is present without ossicular erosion, CWU mastoidectomy is appropriate [22]. For purely extracanalicular mastoid cortex osteomas without secondary cholesteatoma, simple excision via a postauricular approach with cortical mastoidectomy is well established and associated with low long-term morbidity [25,26]. For large extracanalicular osteomas of mastoid cortex origin, combined with middle-ear and mastoid cholesteatoma and ossicular erosion, as in the present case, CWD radical mastoidectomy with meatoconchoplasty provides the most reliable means of disease eradication [12,24]. The principal limitation of this report is the short follow-up of three months. Long-term audiological and imaging data, due at 12–18 months, will be required to confirm disease eradication and assess sensorineural recovery. As a single observation, no generalizable conclusions can be drawn from outcomes alone.

## Conclusion

We report a case of a mastoid cortex osteoma complicated by cholesteatoma confined to the middle ear and mastoid behind a near-intact tympanic membrane, a configuration not previously reported in our review of published case reports. The intraoperative finding of an attic retraction pocket, the absence of any canal-wall cholesteatoma, and the mixed audiometric pattern suggest that Eustachian tube dysfunction may have acted as an independent concurrent mechanism, distinct from the obstructed canal epithelial migration documented in prior reports. Combined HRCT and non-EPI DWI-MRI was essential for preoperative characterization and surgical planning. CWD radical mastoidectomy with meatoconchoplasty provided complete disease eradication at three months. Clinicians should consider middle-ear cholesteatoma via Eustachian tube dysfunction when a temporal bone osteoma presents with mixed hearing loss, even when the tympanic membrane appears structurally preserved.

## Data Availability

Data are available on request from the corresponding author. During the preparation of this manuscript, the authors used AI-based tools to assist with language editing and formatting. All clinical content was reviewed and verified by the authors, who take full responsibility for the final version.
